# Trunk muscle activity during bridging exercises on and off a Swissball

**DOI:** 10.1186/1746-1340-13-14

**Published:** 2005-07-30

**Authors:** Gregory J Lehman, Wajid Hoda, Steven Oliver

**Affiliations:** 1 Department of Graduate Studies, Canadian Memorial Chiropractic College, Toronto, ON, Canada

**Keywords:** EMG, trunk stability, exercise, swiss ball, rehabilitation

## Abstract

**Background:**

A Swiss ball is often incorporated into trunk strengthening programs for injury rehabilitation and performance conditioning. It is often assumed that the use of a Swiss ball increases trunk muscle activity. The aim of this study was to determine whether the addition of a Swiss ball to trunk bridging exercises influences trunk muscle activity.

**Methods:**

Surface electrodes recorded the myoelectric activity of trunk muscles during bridging exercises. Bridging exercises were performed on the floor as well as on a labile surface (Swiss ball).

**Results and Discussion:**

During the prone bridge the addition of an exercise ball resulted in increased myoelectric activity in the rectus abdominis and external oblique. The internal oblique and erector spinae were not influenced. The addition of a swiss ball during supine bridging did not influence trunk muscle activity for any muscles studied.

**Conclusion:**

The addition of a Swiss ball is capable of influencing trunk muscle activity in the rectus abdominis and external oblique musculature during prone bridge exercises. Modifying common bridging exercises can influence the amount of trunk muscle activity, suggesting that exercise routines can be designed to maximize or minimize trunk muscle exertion depending on the needs of the exercise population.

## Background

Trunk muscle co-activation of several muscles is considered necessary in achieving adequate spinal stability to prevent and treat low back injury [1]. Common exercise recommendations from health professionals include trunk exercises to prevent and treat low back injuries. Knowing the trunk muscle activation levels during exercises is important in the prescription and design of exercise programs that aim to increase the training intensity over time (progressive resistance model). Previous research has documented trunk muscle EMG during various exercises designed to train the trunk musculature and during functional activities [2-7]. Ng et al [7] found that abdominal and trunk muscles not only produce torque but also maintain spinal posture and stability during axial rotation exertions. Vera-Garcia et al [8] showed that performing curl-ups on a labile (moveable) surface changes the muscle activity amplitude required to perform the movement. Increases were greatest in the external oblique muscles. Mori [9] documented the trunk muscle activity during a variety of trunk muscle exercises on a Swiss ball. However, comparisons in muscle activity were not made with ground based exercises (no Swiss ball present), therefore, the influence of a Swiss ball on trunk muscle activity compared with ground based bridging is not known.

The importance of trunk muscles in providing adequate spine stability is well established and the role of trunk muscles during a variety of tasks has been well documented. Swiss balls are a common addition to trunk muscle exercises. In fitness centres and rehabilitation centres, Swiss balls are often touted as being superior to ground based exercises in their ability to recruit trunk muscles (rectus abdominis, external oblique, internal oblique, erector spinae). Considering the ubiquity of Swiss balls, one research question was posed: Does the addition of a Swiss ball to bridging exercises influence trunk muscle activity?

The implications of this study are twofold: 1. Modifying trunk muscle activity could be important in the safety and efficacy of rehabilitation exercises when a low level of trunk muscle activity is desired; and 2. Identifying exercises which maximally activate the trunk muscles may make it possible to develop an efficient and less time consuming general strength program that conditions the trunk muscles.

## Methods

### Patient Characteristics and Inclusion Criteria

An all male study population (n = 11, average weight = 85.4 kg (13.1), average height = 179 cm (11) and age 27.6 (3.2) with greater than six months of weight training experience, without back pain or upper limb injuries, was recruited from a convenience sample of College students. Each subject signed an information and consent form, approved by the Research Ethics Board (Canadian Memorial Chiropractic College) explaining the procedures and risks involved with study participation.

### Protocol Overview

The subjects performed five different trunk muscle exercises on two different surfaces (stability ball and floor) and two separate normalization tasks.

### EMG Data Collection Hardware Characteristics

Disposable bipolar Ag-AgCl disc surface electrodes with a diameter of 1.0 cm were adhered bilaterally over the muscle groups studied with a centre to centre spacing of 1.5 cm. EMG electrodes were placed parallel with the muscle fibres on the skin above the rectus abdominus, external oblique, internal oblique and lower erector spinae (L3) on the right side of each subject. The raw EMG was amplified between 1000 and 20,000 times depending on the subject. The amplifier had a CMRR of 10,000:1 (Bortec EMG, Calgary AB, Canada). Raw EMG was banned pass filtered (10 and 1000 Hz) and A/D converted at 2000 Hz using a National Instruments data acquisition system.

### EMG Normalization Procedure

In order to compare values of muscle activity across subjects it was necessary to normalize the EMG data. This required that all EMG values be expressed as a percentage of the maximum EMG activity that can be produced voluntarily by a muscle. Subjects performed two repetitions of two different maximal voluntary contractions (MVC). The subjects were first required to perform a three second maximal isometric trunk curl up and twist against an immovable resistance to maximally recruit the rectus abdominis, internal oblique and external oblique muscles. Second, the subjects performed an isometric prone trunk extension against a fixed resistance to recruit the erector spine and multifidus musculature. The muscle activity during all subsequent experimental tasks was expressed as a percent of the peak activity found during the normalization procedure (MVC exercises). Subjects were allowed to familiarize themselves with the movements before muscle activity was recorded.

### Description of Exercise Tasks

**Figure 1:**
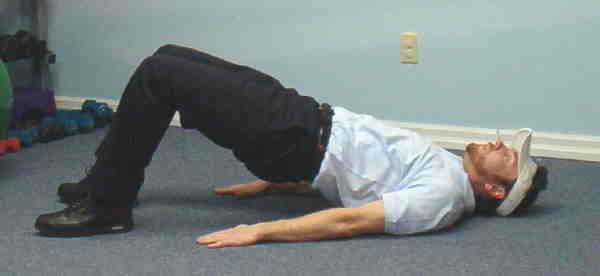
Supine bridge.

**Figure 2:**
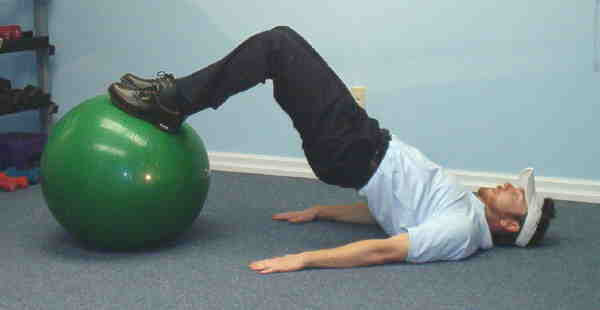
Supine bridge on swiss ball.

**Figure 3:**
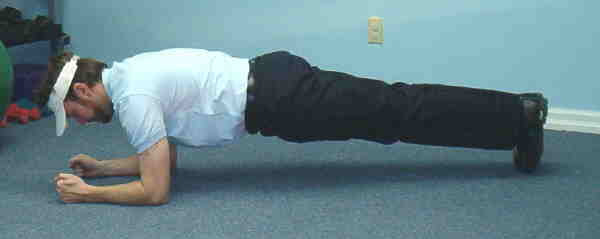
Prone Bridge.

**Figure 4:**
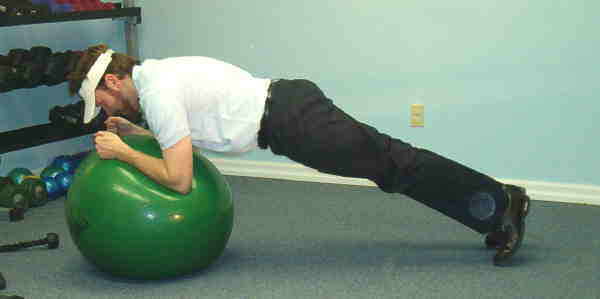
Prone bridge on Swiss ball.

**Figure 5:**
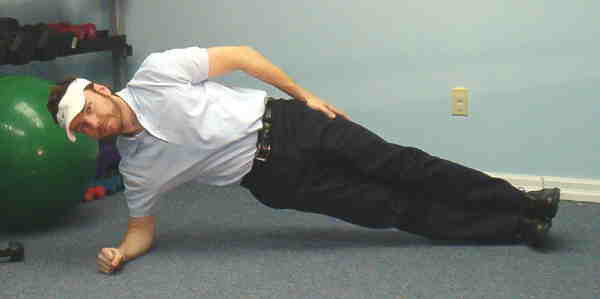
Side bridge.

Feedback from instructors was given in order to achieve a consistent spine and lower limb posture during the following tasks. Subjects aimed to keep their spines in a neutral position with their legs parallel to their trunk during the bridging exercises. The following tasks were chosen because they are common exercises performed in rehabilitation and exercise programs. No attempt was made to control for the different body position relative to gravity between the different exercises. It is recognized that the body's position relative to gravity and the influence of gravity is different between exercises using a Swiss ball and those on the ground. Therefore, conclusions regarding the influence of an unstable surface on trunk muscle cannot be made as the body's position confounds this. The side bridge was added to give the reader a frame of reference for the muscle activity found during the other exercises. It was not performed on the Swiss ball as this exercise is not commonly performed on a Swiss ball and the participants were not familiar with the exercise. Figures 1, 2, 3, 4, 5 illustrate the exercises investigated. Two trials of each of these tasks were recorded. EMG data was collected for 5 seconds during the isometric portion each task. The tasks the participants were required to complete were as follows:

1. Supine Bridge – Subjects began by lying supine on the floor with their feet flat on ground, knees bent 90 degrees, toes facing forward and hands on the floor by their sides, palms facing down. Pushing through the heels, subjects lifted their pelvis off the ground to form a plank.

2. Supine Bridge with Stability Ball – The same procedure was applied as in task #1, however, in this variation the individuals placed their feet flat on a stability ball.

3. Prone Bridge – Subjects assumed a prone position on the floor, and when instructed established a prone plank position with elbows placed beneath the shoulders and upper arms perpendicular to the floor. In this position only the feet and the forearms were touching the floor.

4. Prone Bridge with Stability Ball – The same procedure was applied as in task #3, however, in this variation the individual's forearms were placed on a stability ball.

5. Side Bridge – Subjects assume a side plank position with elbow under shoulder and upper arm perpendicular to the ground.

### EMG Processing

The normalization tasks and the exercise tasks for both studies were processed in an identical manner. Raw EMG from each trial was smoothed using an RMS averaging (window of 100 ms, 50 ms overlap) technique. The average activity, expressed as a percent of the normalization contraction, was found for the exertion portion of each exercise and repetition. The average of two repetitions for each exercise and for each muscle was then calculated.

### Statistical Analysis

A repeated measures ANOVA with a post hoc Tukey test was used to determine activation level differences within each muscle across bridging exercise tasks.

All statistical tests were performed at the 5% level of significance.

## Results

**Figure 6:**
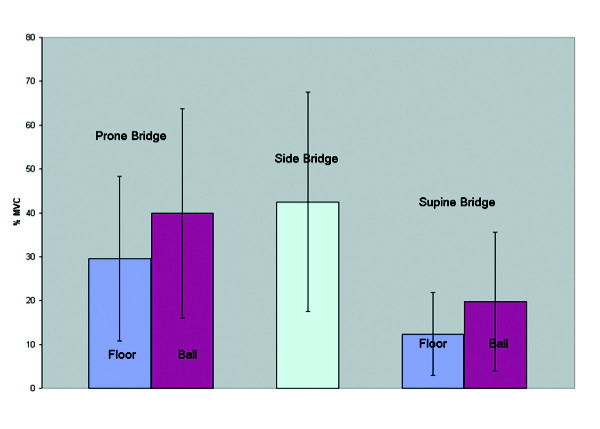
Internal oblique group average activity on/off a Swiss ball during bridging exercises.

**Figure 7:**
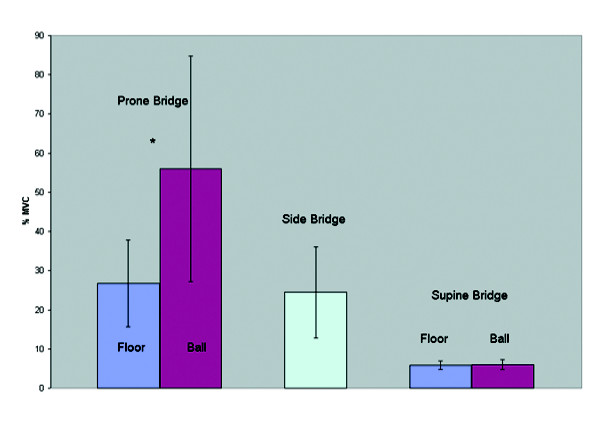
Rectus abdominis group average activity on/off a Swiss ball during bridging exercises.

**Figure 8:**
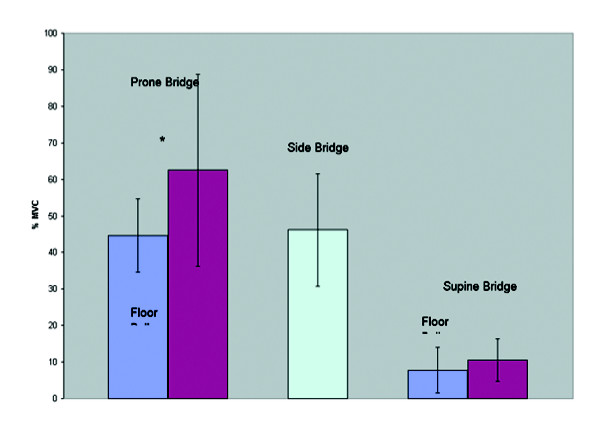
External oblique group average activity on/off a Swiss ball during bridging exercises.

**Figure 9:**
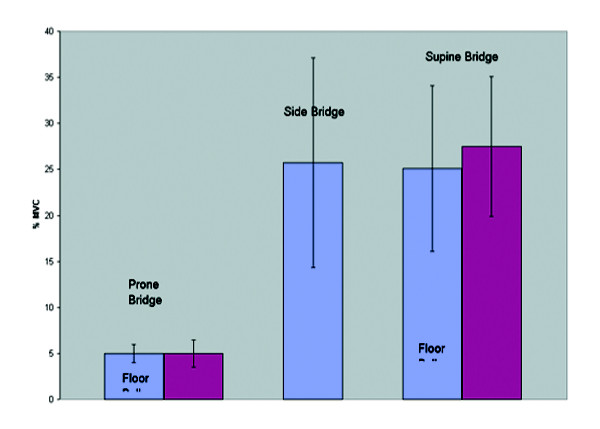
Erector spinae group average activity on/off a Swiss ball during bridging exercises.

Table 1 depicts the muscle activation levels across exercises. The addition of an exercise ball did not influence the muscle activity in the Internal Oblique (Figure 6) in both bridging exercises. During the prone bridge the addition of an exercise ball resulted in increased myoelectric activity in the rectus abdominis and external oblique (Figure 7 and Figure 8). The exercise ball did not influence the Rectus Abominis or the External Oblique muscle activity during a supine bridge. The addition of an exercise ball did not influence the Erector Spinae (Figure 9) activity during the supine bridge or the prone bridge.

**Table 1:** Muscle activation levels expressed as percentage of the Maximum Voluntary Contraction for bridging exercises on different surfaces.

Column #	1	2	3	4	5
**Exercise**	**Pr Br Floor**	**Pr Br Ball**	**Side Bridge**	**Su Br Ball**	**Su Br Floor**
**IO Avg**	29.5	39.8	42.5	19.7	12.3
*Stdev*	*18.8*	*23.9*	*25.2*	*15.8*	*9.5*
Different From*	-	4,5	4,5	2,3	2,3
					
**RA Avg**	26.6	55.9	24.4	6.05	5.84
*Stdev*	*11.1*	*28.8*	*11.7*	*1.3*	*1.1*
Different From*	2,4,5	1,3,4,5	2,4,5	1,2,3	1,2,3
					
**EO Avg**	44.6	62.5	46.1	10.6	7.8
*Stdev*	*14.8*	*26.3*	*15.4*	*5.7*	*6.3*
Different From*	2,4,5	1,3,4,5	2,4,5	1,2,3	1,2,3
					
**ES Avg**	4.98	5.00	25.7	27.4	25.01
*Stdev*	*1.05*	*1.46*	*11.3*	*7.56*	*9.02*
Different From*	3,4,5	3,4,5	1,2	1,2	1,2

* This row indicates which other exercise the myoelectric signal for the respective column is statistically different (p < .05) from. (Avg = Average muscle activity in % MVC, Stdev = Standard deviation, IO = Internal Oblique, RA = Rectus Abdominis, EO = External Oblique, ES = Erector Spinae. Pr Br = Prone Bridge, Su Br = Supine Bridge.

The side bridge produced the highest myoelectric activity in both the Internal Oblique and Erector Spinae. The prone bridge with arms on a Swiss ball produced the highest myoelectric activity in both the Rectus Abdominis and External Oblique.

## Discussion

The primary aim of this study was to determine if performing bridging exercises on a Swiss ball rather than the ground resulted in increases in trunk muscle activity. A blanket statement that a more labile surface (the Swiss ball) increases trunk muscle activity cannot be made. The influence of surface stability on muscle activity appears to be muscle and exercise dependent. For example, during the prone bridge the primary mover (the rectus abdominis resisting trunk extension) was the most influenced by the addition of a Swiss ball. Conversely, during a supine bridge, one of the primary movers, the erector spinae, was not influenced by surface stability (the other primary mover, the Gluteus Maximus was not studied). It may be argued that the increase in activation levels of the external oblique and the rectus abominis during prone bridging appear to be caused by decreases in surface stability and not different biomechanical demands due to the body's position relative to gravity. This finding agrees with the Vera-Garcia et al [8] study that investigated trunk curl up exercises. While there were differences in the body's position relative to gravity between the ground exercise and the ball exercise during prone bridging, performing the bridge on a ball finds the participant in a more vertical position. This suggests there is less force creating a trunk extension movement (i.e. gravity attempts to increase lordosis which is resisted by muscle activity) due to the fact that the centre of mass of the trunk and head segment would be closer to the axis for trunk extension. Therefore less muscle activity may have been generated to resist this torque (compared with the ground based bridge) and more muscle activity may have been required to produce secondary spinal stabilization due to the labile surface.

**Figure 10:**
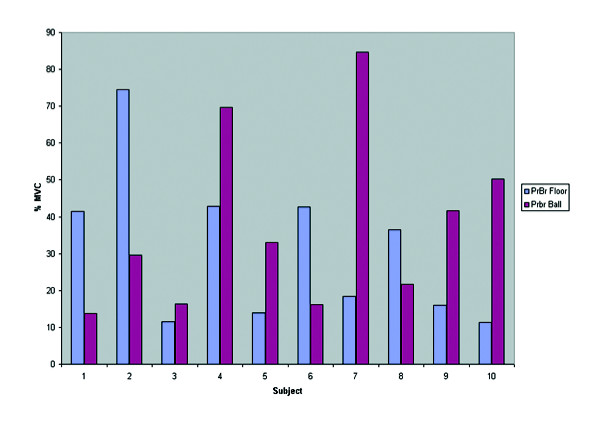
Internal oblique muscle activity for each participant during one repetition of prone bridging on/off a Swiss ball.

An important observation from all exercise tasks was the large variability in muscle activity between subjects that can greatly influence the interpretation of these results. Figure 10 illustrates an example of this variability. Figure 6 shows the average activity in the internal oblique muscle during prone bridging on and off a Swiss ball for one repetition from each subject. This indicates that some subjects showed large changes in muscle activity while others showed minimal changes when modifications to the exercise tasks were made. It is possible that some subjects volitionally contracted their trunk muscles to provide stability while some others may have not. It is possible that individuals may be able to influence their trunk muscle activity either through verbal encouragement, or feedback produced by electromyography. Additionally, the variability may have been due to slight variations in participant posture or task performance. While exercise standardization was sought through verbal correction of form, it is possible that differences in task performance between the subjects still occurred. Further research may wish to determine the influence of electromyographic feedback on influencing the trunk activation levels during resistance exercise. This may decrease the variability between subjects.

This study is limited because it only measured the trunk muscle activity during the various exercises. No measurements were made nor a biomechanical model constructed to determine the compressive or shear loading on the spine during the tasks. This type of kinematic, and subsequently force data, is optimal when determining the safety and tissue loading properties of various movements. Also, this study did not quantitatively measure spinal posture. This may influence the muscle activation levels. While a consistent spinal posture was encouraged and monitored by the experimenters, it is also possible that minor differences in spine posture did occur. Monitoring spinal posture via a kinematic measurement system (eg. Electromagnetic tracking) may be important in future work.

While increased trunk muscle activation can result in higher compressive loads on the spine [12], is this amount of trunk muscle activity necessarily increasing the risk of injury? We are unable to say if increases in activity levels are due to biomechanical demands, or if they are due to motor control decisions that permit enhancements in spine stability that may decrease the risk of injury. Conversely, if an exercise modification results in decreases in muscle activity, is this always beneficial in terms of injury prevention? Is it possible that subjects who lower their muscle activation levels during tasks predispose themselves to a "buckling" type injury because sufficient spinal stability is not created with the current amount of trunk muscle activity [13]? It is important to note that this study measured trunk muscle activity in an athletic young homogenous population. Sedentary individuals or those with trunk or lower leg injuries may show different results.

## Conclusion

Differences in trunk muscle activity are seen with the addition of a Swiss ball to bridging exercises. It cannot be concluded that these differences are solely due to changes in surface stability due to the different biomechanical demands of the exercises. Future research should control for exercise posture to determine how surface stability influences muscle activity.

## Competing interests

There are no competing interests for this research project.

Participants read and signed an information and consent form approved by the Research Ethics Board (Canadian Memorial Chiropractic College). The study protocol was approved by the Research Ethics Board.

## Authors' contributions

GJL; study conception, study design, data collection, statistical analysis, manuscript preparation. WH & SO: study design, data collection, data processing.
